# Forest topsoil salvage and placement depth affects oil sands reclamation in the boreal forest

**DOI:** 10.1371/journal.pone.0306018

**Published:** 2024-07-31

**Authors:** Dean D. Mackenzie, M. Anne Naeth

**Affiliations:** 1 Vertex Resource Group Ltd., Sherwood Park, Alberta, Canada; 2 Department of Renewable Resources, University of Alberta, Edmonton, Alberta, Canada; USDA Forest Service Southern Research Station, UNITED STATES

## Abstract

Reclamation of disturbances from oil sands mining requires effective soil management to ensure successful plant establishment and to promote recovery of native plant communities. In this study we investigated the effects of salvage depths (shallow vs. deep) and placement depths (shallow vs. deep) of forest topsoil on plant establishment, species richness, and soil properties in two substrate types (sand and peat-mineral). Shallow salvage led to greater tree stem densities and higher canopy cover for most plant groups, although there was no significant difference in species richness between shallow and deep salvages. Deep placement generally resulted in greater canopy cover, while its effect on plant density was very small for most plant groups. On peat-mineral substrate, fewer differences were detected between shallow and deep salvage, and multiple treatments resulted in greater cover. Findings suggest that a balance between maximizing the area over which propagules are redistributed and providing sufficient resources for successful plant establishment is necessary. Forest topsoil from shallow salvages and deep placements is recommended when targeting increased site productivity and species diversity. In contrast, deep salvage should be used when the primary objective is to obtain maximum reclamation material volume. Salvage depth effects may be influenced by substrate type, with peat-mineral substrate providing more favourable conditions for plant establishment. Further research is needed to assess the long-term impacts of different salvage and placement depths on plant community development and the potential effects of substrate properties on soil and plant response.

## Introduction

Oil sands mining where vegetation and overburden are removed to expose underlying bitumen [1] has disturbed approximately 1,055 km^2^ of natural boreal forest in northern Alberta [2]. According to provincial regulatory requirements, these disturbances must be reclaimed to diverse, self-sustaining plant communities similar to the surrounding region [3]. Salvaging, which in this context refers to the careful removal and preservation of boreal forest topsoil (forest floor material mixed with upper soil mineral horizons), provides industry with a means to use a local diverse seed source and fertile surface soil to meet reclamation requirements [4–6]. Forest floor materials and topsoil are important sources of forest understory propagules for upland forest reclamation [5, 7]. Direct placement of forest topsoil is considered a best practice in the oil sands [3, 8, 9].

The area available for forest topsoil salvage in the Athabasca Oil Sands Region is limited and much of it is developed on coarse texture, nutrient poor and rapidly drained soil [10]). *Pinus banksiana* Lamb. (jack pine) forests dominate the dryer soils in the study area, with *Populus tremuloides* Michx. (trembling aspen) dominant on moister sites. Fewer seeds have been found in *Pinus banksiana* soils than in forests on fine texture soils [5, 11–14] and forests developed on coarse texture soil are less fertile than those from fine texture soils [15]. How successful these soils will be in providing propagules for revegetation and sustaining plant growth on post-disturbed landscapes is not well understood. The limited availability of these materials requires a strategic use that balances sufficient quantities to support self-sustaining diverse plant communities across large landscapes.

Plant species establishment from forest topsoil is dependent on salvage depth. Deep salvage, which involves removing a thicker layer of soil, typically greater than 10 cm, provides more volume to spread across a larger landscape. Most propagules in boreal forests are in the forest floor and the upper few cm of mineral soil [13, 16, 17], decreasing with increasing depth [18–22]. Thus, deep salvage of the forest floor and soil dilutes propagules in the surface of the replaced materials relative to shallow salvage. Soil nutrients and organic matter vary with depth, with organic layers containing more organic matter, available nutrients and cation exchange sites than mineral horizons [23, 24]. With increasing depth, available nutrients and organic matter decrease and exchangeable cations increase. While there have been some studies examining the effects of soil salvage depth on forest soils, such as Mackenzie and Naeth [5] and Macdonald et al. [25], the overall understanding of this relationship remains limited.

Placement depth of forest topsoil during reclamation will affect plant species establishment and availability. Many studies have recommended efficient use of topsoil through shallow placement, typically around 10 cm, since most seeds emerge from the near surface [26–28]. In contrast, Mackenzie and Naeth [5] found deep placement, which involves spreading forest topsoil in a thicker layer typically around 20 cm or greater, of forest topsoil developed on fine texture soils provided more species, greater densities and higher plant cover then shallow placement. Deep applications contained greater organic carbon and nutrients then shallow applications. MacKenzie and Naeth [5] did not attribute their results to application depth but to increased fine particle material in the upper 10 cm admixed during placement using large equipment.

Several substrates are available in the Athabasca Oil Sands Region, including peat-mineral mix and fine (loam to fine texture) and coarse texture (sand) parent material (mineral). Applying forest topsoil at shallow depths may be acceptable if a substrate can provide soil water and nutrients for successful establishment. Optimizing placement depth will help reduce waste of the valuable forest floor material.

This research was conducted to determine effectiveness of forest topsoil salvaged from coarse texture soils in providing an alternative cover soil and in situ propagules for revegetation of mined oil sands than the commonly used peat-mineral mix. The effect of salvage depth, application depth and substrate type on plant establishment and soil properties at on operational scale were evaluated.

## Materials and methods

### Research site description

The research was conducted north of Fort McMurray, Alberta, Canada, at the Syncrude Canada Ltd mine (latitude 57° 21’ N, longitude 111° 31’ W) in the central mixed wood subregion of the boreal natural region [29]. Climate is cool temperate with short, cool summers and long, cold winters [30]. Mean annual temperature is 0.3 ºC. The 1944 to 2007 long term mean annual precipitation was 471.2 mm, approximately 322.7 mm as rain and 148.5 cm as snow [31].

Soils are Histosols (organic) in lowlands and Eluviated and Eutric Cambisols (mineral) in uplands [10]. The organic soils have thick organic horizons (O horizons) overlaying coarse textured mineral layers or other organic layers. The mineral soils have a thin forest floor layer overlying a coarse textured mineral horizons. Pre-disturbance vegetation was representative of the mixed wood boreal forest. Undisturbed organic soils are dominated by *Picea mariana* (P.) Mill. (black spruce) and *Larix laricina* (Du Roi) K. Koch (tamarack), and mineral soils by coniferous-deciduous forest. Uplands typically consist of *Pinus banksiana*, *Populus tremuloides* and *Picea glauca* (Moench) Voss (white spruce) [1].

The 25 ha donor site was a 50 year old forest, harvested of timber in the summer, 4 to 6 months before salvage. Topography was gently to strongly undulating. Vegetation was dominated by *Pinus banksiana* and *Populus tremuloides* with a diverse understory of shrubs and herbaceous species. One area had an overstory of *Pinus banksiana*, the other a mix of *Pinus banksiana* and *Populus tremuloides*. Mean forest floor depth was 5 cm, ranging from 2 cm in *Pinus banksiana* stands to 8 cm in mixed stands.

Forest topsoil was salvaged at 10 (shallow salvage) and 25 cm (deep salvage) in September (prior to summer assessment of the plots the following year) using a D7 Caterpillar crawler tractor. Salvaged material was stored in small windrows (2 to 3 m high, 4 to 6 m wide) for five months until placement.

Field site access was approved by Syncrude Canada Ltd. No formal permits were required for access as this was approved for each site access with a check in and check out policy. Strict safety training was required and all company driving protocols were followed.

### Experimental design

Two independent experimental sites were established, 350 m apart, on lower slopes of a north facing, lean oil sands (bitumen content insufficient for economic extraction) overburden dump. At one site, 1 m of sand was placed on overburden (soil overlying the oil sands deposit); at the other site, 1 m of mixed 50% sand and 50% fen peat (peat-mineral mix) was placed on overburden. Slope was 10 to 20% on sand substrate and 5 to 10% on peat-mineral substrate. A complete randomized design at each site consisted of 10 and 25 cm salvage depths of topsoil and 10 (shallow) and 20 cm (deep) placement depths of forest topsoil. A treatment consisting of no topsoil placement was located only on the peat-mineral substrate. A no topsoil treatment could not be established on sand substrate due to limited space and high erosion potential of exposed sand. Each treatment was replicated three times; each replicate was 15 by 70 m to accommodate operational scale equipment. A mid-sized bulldozer (D7) was used for the salvage and placement operations, and a large capacity haul truck (300 tonne) was used for transporting the topsoil.

### Measurements

Vegetation was assessed in mid July of year 1 and year 3 of the study. Ten randomly located 1 m^2^ quadrats were placed in each of upper, mid and lower slope positions in each treatment replicate. Plant density and ocular canopy cover by species were determined in each quadrat in year 1; ocular canopy cover by species and woody stem density were determined in the following years. Species nomenclature followed Moss [32].

Soils were sampled in August of year 3 of the study. In each treatment replicate, 5 random subsamples were taken in each of 3 slope positions and composited into a polyethylene bag, for a total of 3 samples per replicate. All treatments were sampled with a shovel to corresponding placement depths.

Soils were analyzed according to Carter [33] unless otherwise noted. Saturation %, pH, electrical conductivity, sodium adsorption ratio, soluble cations (calcium, potassium, magnesium, sodium) and soluble anions (chloride, sulfate) were determined from saturated paste extract. Total nitrogen was analyzed by digestion after treatment with Devarda’s alloy to convert nitrate to ammonium. Total carbon was determined by combustion. Extractable cations (calcium, potassium, magnesium, sodium) and cation exchange capacity were determined with ammonium acetate at pH 7.0. Available phosphorus and potassium were determined using modified Kelowna extraction. Available nitrate was extracted with 2 molar potassium chloride.

### Statistical analyses

Species were categorized into 6 plant groups based on morphology (tree, shrub, forb, grass, sedge, lily) and one plant group with a sum of all plants (total). Unknown monocotyledons and dicotyledons were only included in the total. Subsample data, including slopes, within each experimental unit were averaged to give one value per experimental unit for each variable. Species richness was calculated by totalling number of species per experimental unit or replicate. Diversity was calculated for each experimental unit as the Shannon-Wiener index (H’) and evenness using the formula E = H’/log10R [34]. Unidentified plants (2 to 5 per experiment) were excluded from diversity measures. Density data were presented as plants m^-2^.

Analyses were conducted using analysis of variance (ANOVA) in SPSS 18.0. Each experimental site was analyzed separately. While this study presents descriptive observations of the differing responses between forest topsoil treatments on sand substrates versus peat-sand substrates it does not include a formal statistical comparison. Two way fixed effects ANOVA was used to determine effects of salvage depth and placement depth, excluding the no topsoil placement depth on the peat-mineral substrate. Significant interaction effects in two way ANOVA were analyzed comparing forest topsoil treatments using one way ANOVA, if main effects were significant, differences among treatments were further analyzed using LSD. One way fixed effects ANOVA was used to determine significant differences between forest topsoil treatments and no topsoil depth treatment on the peat-mineral substrate [35]. Significant main effects using one way ANOVA were further analyzed using least squares difference (LSD) post hoc test for significant differences between control and forest topsoil treatments [36]. The percent cover response variables for the grass plant group were transformed to meet assumptions of normality based on the Shapiro-Wilk test. Due to non-normality, the analysis excluded available nitrate and evenness; however, the mean values are still presented. Significance effects were evaluated at p ≤ 0.05.

## Results

### Vegetation

Over 3 years, 65 plant species were found; 61 in forest topsoil and 41 in no topsoil treatments on the peat-mineral control treatments. Species richness increased over time and was significantly greater in forest topsoil each year (year 1 p ≤ 0.002, year 3 p ≤ 0.035) than in peat-mineral substrate (Table 1). Diversity did not significantly differ (p ≥ 0.05) between forest topsoil and no topsoil treatment on the peat-mineral substrate; however, diversity was greater in forest topsoil. Salvage and placement depth had little effect on diversity (Table 1). In year 3, species richness was greater with deep salvage than shallow.

**Table 1:** Mean diversity measures for forest topsoil treatments on sand substrate, forest topsoil and control treatments on peat-mineral substrate.

Year	Salvage Depth (cm)	Placement Depth (cm)	Moss						Peat-Mineral					
			Richness		Diversity		Evenness		Richness		Diversity		Evenness	
1	10	10	21.7	(1.20)	1.52	(0.45)	0.49	(0.14)	21.7^*^	(2.96)	1.70	(0.26)	0.60	(0.12)
		20	22.7	(0.33)	1.11	(0.23)	0.36	(0.08)	21.7^*^	(1.20)	2.14	(0.35)	0.70	(0.11)
	25	10	21.7	(0.33)	1.31	(0.20)	0.43	(0.06)	22.3^*^	(1.33)	2.07	(0.28)	0.70	(0.10)
		20	21.7	(1.45)	1.60	(0.04)	0.52	(0.03)	21.7^*^	(2.03)	1.90	(0.28)	0.60	(0.09)
	No topsoil		-		-		-		9.3	(0.33)	1.43	(0.08)	0.60	(0.04)
3	10	10	23.0	(1.00)	2.25	(0.12)	0.72	(0.03)	24.3^*^	(2.60)	1.86	(0.05)	0.59	(0.03)
		20	23.7	(0.33)	2.19	(0.09)	0.69	(0.03)	25.3^*^	(0.33)	2.06	(0.15)	0.64	(0.05)
	25	10	27.0	(2.52)	2.16	(0.05)	0.66	(0.03)	30.0^*^	(3.79)	2.20	(0.01)	0.65	(0.02)
		20	25.7	(2.40)	2.20	(0.17)	0.68	(0.03)	27.0^*^	(2.08)	2.07	(0.11)	0.63	(0.05)
	No topsoil		-		-		-		17.7	(0.88)	1.63	(0.36)	0.56	(0.12)

Data are mean and (standard error), n = 3. In columns * denotes forest topsoil treatments significantly different from the control at p ≤ 0.05.

Pioneer species such as *Urica dioica* L. and *Geranium bicknellii* Britt had a reduced canopy cover after 3 years while most woody plants increased in abundance in forest topsoil treatments. *Urtica dioica* increased in canopy cover in the no topsoil treatment on the peat-mineral substrate. *Sonchus arvense* L. abundance, a noxious weed as per the Alberta Weed Control Act [37], decreased over time in forest topsoil treatments on sand substrate and increased in all treatments in peat-mineral substrate. *Populus tremuloides* Michx. increased in abundance in all forest topsoil treatments after 3 years and decreased in abundance in the peat-mineral treatment. Many dry land species in forest topsoil appeared in peat-mineral substrate in year 3 from forest topsoil treatments via seed dispersal and vegetative expansion. Species such as *Achillea millefolium* L. (common yarrow), *Amelanchier alnifolia* (Nutt.) (saskatoon berry), *Arctostaphylos uva-ursi* (L.) Spreng. (kinnikinnick), *Carex siccata* Dewey (hay sedge), *Carex aenea* Fern. (bronze sedge), *Fragaria virginiana* Duchesne (strawberry), *Pinus banksiana*, and *Vaccinium myrtilloides* found in the peat-mineral treatment were usually close to forest topsoil plot edges (seed and vegetative expansion). Some species in peat-mineral plot centers in year 3 likely established from seed from parent plants (*Achillea millefolium*) as noted by seedling proximity to parent plants, or eroded by wind and water from forest topsoil plots onto peat-mineral plots (*Pinus banksiana*).

Density of most herbaceous plants was not significantly different (p ≥ 0.05) with peat-mineral and forest topsoil; 20 cm forest topsoil placement had greater (p ≤ 0.002) lily (Table 2). Shrub density increased over time, significantly greater in forest topsoil treatments than no topsoil on peat-mineral substrate each year (year 1 p ≤ 0.007, year 3 p ≤ 0.0004) (Table 3). Tree density was greater in forest topsoil treatments each year; only 10 cm salvage with 20 cm placement was significantly greater (p ≤ 0.021) than no topsoil treatment on peat-mineral substrate by year 3. Tree density in year 3 was significantly greater on shallow salvage treatments (p ≤ 0.03), and thick placement depths had significantly greater shrub densities (p ≤ 0.009). Density of most herbaceous plant groups in year 1 was not affected by salvage or placement depth (Table 2). Shallow salvage had significantly greater grass density (p ≤ 0.007) on sand. There was a significant interaction effect for lily density (p ≤ 0.022) on peat-mineral substrate; shallow salvage and deep placement had significantly greater density than other treatments and shallow salvage, shallow placement had lowest densities. Shallow salvage and placement had lowest shrub stem densities on peat-mineral substrate (Table 4).

**Table 2:** Mean density (plants m^-2^) in year 1 for herbaceous plant groups on sites with forest topsoil on sand and peat-mineral substrates.

Substrate	Salvage Depth (cm)	Placement Depth (cm)	Total		Forb		Grass		Sedge		Lily	
Sand	10	10	12.1	(0.63)	3.5	(0.40)	1.1^a^	(0.21)	1.0	(0.10)	1.1	(0.21)
		20	11.7	(1.44)	3.2	(0.33)	0.8^a^	(0.09)	0.9	(0.21)	0.8	(0.08)
	25	10	10.7	(1.32)	5.5	(1.45)	0.4^b^	(0.06)	0.7	(0.04)	0.4	(0.06)
		20	13.0	(2.53)	5.5	(1.87)	0.5^b^	(0.15)	0.9	(0.26)	0.5	(0.15)
Peat-Mineral	10	10	7.5	(1.71)	3.0	(1.05)	0.5	(0.09)	1.3	(0.16)	0.1^c^	(0.03)
		20	8.8	(0.97)	2.2	(0.43)	0.3	(0.09)	1.1	(0.12)	0.9^*a^	(0.21)
	25	10	8.4	(3.00)	3.8	(1.77)	0.4	(0.12)	1.1	(0.44)	0.3^b^	(0.08)
		20	9.6	(1.82)	4.6	(1.09)	0.3	(0.05)	0.6	(0.15)	0.4^*b^	(0.11)
		No topsoil	4.7	(1.36)	3.0	(0.48)	0.7	(0.31)	0.9	(0.65)	0.0	-

Data are mean and (standard error), n = 3. In columns * denotes forest topsoil treatments significantly different from the control. In columns different letters denotes significant differences for two way ANOVA. Significant differences at p≤0.05.

**Table 3:** Mean tree and shrub density (stems m^-2^) in year 1 on sites with forest topsoil on sand and peat-mineral substrates.

Year	Salvage Depth (cm)	Placement Depth (cm)	Sand				Peat-Mineral			
			Trees^1^		Shrubs		Trees		Shrubs	
1	10	10	0.7	(0.19)	5.3	(0.92)	0.1	(0.07)	2.6^*^	(0.37)
		20	1.5	(0.74)	4.9	(0.74)	0.3	(0.08)	4.0^*^	(0.80)
	25	10	0.5	(0.09)	3.4	(0.68)	0.2	(0.11)	2.5^*^	(0.59)
		20	0.4	(0.12)	5.3	(0.49)	0.2	(0.02)	3.4^*^	(0.77)
3	10 cm	10	0.5^a^	(0.19)	6.4^b^	(0.63)	0.1	(0.07)	3.3^*^	(0.44)
		20	1.2^a^	(0.40)	8.8^a^	(1.09)	0.4^*^	(0.10)	6.2^*^	(1.09)
	25	10	0.3^b^	(0.12)	4.9^b^	(0.75)	0.1	(0.06)	5.0^*^	(1.03)
		20	0.3^b^	(0.08)	8.0^a^	(0.65)	0.1	(0.04)	4.3^*^	(0.92)
	No topsoil						0.02	(0.01)	0.7	(0.49)

Data are mean and (standard error), n = 3. In columns * denotes forest topsoil treatments significantly different from the control. In columns different letters denotes significant differences for two way ANOVA. Significant differences at p≤0.05.

**Table 4:** Mean canopy cover in year 3 for plant groups on sites where forest topsoil was placed on sand and peat-mineral substrate.

	Salvage Depth (cm)	Placement Depth (cm)	Total		Trees		Shrubs		Forb		Grass		Sedge	
Sand	10 cm	10 cm	18.64^b^	(2.47)	1.04	(0.77)	3.56^bc^	(0.45)	7.88	(0.30)	3.54^a^	(0.57)	2.59	(1.69)
		20 cm	49.00^a^	(4.16)	2.66	(0.20)	17.81^a^	(1.18)	12.41	(1.15)	3.84^a^	(0.22)	12.26	(4.40)
	25 cm	10 cm	19.34^b^	(3.41)	0.33	(0.13)	2.36^c^	(0.81)	13.76	(4.34)	1.77^b^	(0.67)	1.10	(0.23)
		20 cm	23.50^b^	(3.44)	0.36	(0.04)	5.31^b^	(1.17)	14.73	(1.67)	1.57^b^	(0.17)	1.46	(0.96)
Peat -	10	10	32.46^*Ab^	(2.33)	0.31	(0.27)	5.83^*^	(2.26)	15.03^*^	(4.11)	1.96^a^	(0.21)	9.33^*a^	(2.45)
mineral		20	47.70^*Aa^	(7.18)	0.77	(0.50)	11.21^*a^	(3.64)	20.72^*^	(3.09)	3.55^a^	(0.94)	11.41^*a^	(2.48)
	25	10	20.41^*Bb^	(2.94)	0.14	(0.08)	2.94^*^	(1.08)	14.64^*^	(2.66)	1.00^*b^	(0.17)	1.66^*b^	(0.08)
		20	32.26^*Ba^	(7.52)	0.05	(0.04)	4.10^*^	(0.57)	24.94^*^	(7.56)	1.09^*b^	(0.25)	2.04^*b^	(0.66)
	No topsoil		7.24	(1.32)	0.02	(0.01)	0.68	(0.49)	2.68	(0.74)	3.22	(1.97)	0.64	(0.35)

Data are mean and (standard error), n = 3. SD, salvage depth; PD, placement depth. In columns different letters denote significant differences at p≤0.05. In columns * denotes forest topsoil treatments significantly different from the control at p≤0.05.

Canopy cover of most plant groups on forest topsoil was significantly greater than no topsoil on peat-mineral substrates for most plant groups, including total (p ≤ 0.002), shrub (p ≤ 0.008), forb (p ≤ 0.038), grass (p ≤ 0.040) and sedge (p ≤ 0.001) (Table 4). Lily plants were not found in peat-mineral controls. Generally, shallow salvage treatments resulted in greater canopy cover for most plant groups. Significant interaction effects were found for total cover (p ≤ 0.005) and shrub cover (p ≤ 0.001) on sand substrate; shallow salvage and deep placement had greatest canopy cover. Shallow salvage resulted in significantly greater grass cover (p ≤ 0.003) on sand substrate. Shallow salvaged forest topsoil placed on peat-mineral substrate provided significantly more canopy cover for total (p ≤ 0.038), grass (p ≤ 0.001) and sedge (p ≤ 0.001) plant groups. Placement depth had more effect with forest topsoil on sand than on peat-mineral substrate; however, deep placement resulted in significantly greater total canopy cover (p ≤ 0.040). Grass in peat-mineral substrate was significantly greater than in 25 cm salvage.

### Soil properties

Organic carbon was lowest in forest topsoil on sand. Shallow placed forest topsoil on peat-mineral substrate had soil properties similar to those of the peat-mineral substrate. Soil properties varied among treatments with significantly higher pH (p ≤ 0.001), electrical conductivity (p ≤ 0.001), sodium adsorption ration (p ≤ 0.008), total carbon (p ≤ 0.046), total nitrogen (p ≤ 0.050), CEC (p ≤ 0.049), nitrate (p ≤ 0.030) and sulphur (p ≤ 0.0001) in peat-mineral relative to forest topsoil treatments (Table 5). No available phosphorus was detected in peat-mineral substrate. Effects of forest topsoil salvage and placement depth on chemical properties varied with substrate, with shallow salvages on sand generally having highest values of most properties (Table 5). Shallow salvage had significantly greater total carbon (p ≤ 0.001) and total nitrogen (p ≤ 0.015) on sand substrate and less phosphorus on peat-mineral substrate (p ≤ 0.001). Shallow placement had significantly greater pH (p ≤ 0.01), electrical conductivity (p ≤ 0.005), sodium adsorption ratio (p ≤ 0.001) and sulphur (p ≤ 0.002) on peat-mineral substrates and pH (p ≤ 0.039) and sulphur on sand substrate (p ≤ 0.011). Significant interaction effects (p ≤ 0.005) were found for cation exchange capacity on sand; shallow salvage deep placement had the greatest cation exchange capacity and deep salvage deep placement has the lowest.

**Table 5:** Mean values of chemical parameters from forest topsoil and controls placed on sand and on peat-mineral substrates.

Parameter	Sand				Peat-Mineral				
	10/10	10/20	25/10	25/20	10/10	10/20	25/10	25/20	No topsoil
pH	5.94^a^	5.44^b^	5.82^a^	5.64^b^	6.47^*a^	6.00^*b^	6.37^*a^	5.89^*b^	7.4
	(0.11)	(0.18)	(0.14)	(0.11)	(0.15)	(0.14)	(0.12)	(0.16)	(0.03)
EC	0.40^a^	0.30^a^	0.28^b^	0.22^b^	1.02^*a^	0.66^*b^	1.44^*a^	0.54^*b^	2.59
(dS/m)	(0.07)	(0.03)	(0.04)	(0.01)	(0.12)	(0.16)	(0.26)	(0.06)	(0.11)
SAR	0.27	0.38	0.33	0.34	0.16^b^	0.24^*a^	0.12^b^	0.31^*a^	0.17
	(0.02)	(0.06)	(0.03)	(0.03)	(0.01)	(0.02)	(0.03)	(0.04)	(0.03)
Total carbon	1.14^a^	1.41^a^	0.74^b^	0.68^b^	1.22^*^	1.13^*^	1.02^*^	0.79^*^	14.52
(%)	(0.14)	(0.15)	(0.07)	(0.04)	(0.23)	(0.08)	(0.31)	(0.18)	(1.69)
Total nitrogen	0.04^a^	0.05^a^	0.03^b^	0.03^b^	0.06^*^	0.04^*^	0.04^*^	0.03^*^	0.51
(%)	(0.01)	(0.01)	(0.00)	(0.00)	(0.01)	(0.00)	(0.01)	(0.01)	(0.09)
CEC	3.24^b^	4.09^a^	3.11^b^	2.72^c^	3.46^*^	3.46^*^	3.38^*^	3.13^*^	13.59
(meq/100g)	(0.16)	(0.41)	(0.14)	(0.06)	(0.42)	(0.07)	(0.66)	(0.07)	(1.46)
Available Nutrients (mg kg-1)									
Nitrate	0	0.12	0	0	0.8^*^	0.34^*^	0.00^*^	0.23^*^	2.07
	(0.00)	(0.12)	(0.00)	(0.00)	(0.88)	(0.18)	(0.00)	(0.23)	(0.24)
Phosphorus	13.44	16.56	20.11	22.44	19.44*^b^	22.56*^b^	31.89*^a^	31.44*^a^	0
	(2.78)	(2.63)	(2.26)	(6.63)	(3.16)	(2.00)	(0.62)	(0.97)	(0.00)
Potassium	29.78	33.89	29.33	26.67	20.44	26.56	24.4	32.33	21.56
	(2.21)	(2.42)	(3.47)	(3.79)	(1.31)	(1.16)	(3.18)	(5.29)	(2.75)
Sulphur	6.44^a^	3.89^b^	4.56^a^	2.78^b^	40.78^*a^	26.33^*b^	100.78^*a^	20.56^*b^b	595.67
	(1.78)	(0.40)	(0.48)	(0.73)	(4.92)	(9.79)	(21.25)	(2.95)	(130.09)

Data are mean and (standard error), n = 3. 10/10 = 10 cm salvage depth, 10 cm placement depth; 10/20 = 10 cm salvage depth, 20 cm placement depth; 25/10 = 25 cm salvage depth, 10 cm placement depth; 25/20 = 25 cm salvage depth, 20 cm placement depth. In columns different letters denote significant differences, where upper case letters denote a significant difference between salvage depth and lower case letters denote a significant difference between placement depth or for forest topsoil treatments where interaction effects are significant at p≤0.05. EC = electrical conductivity, SAR = sodium adsorption ratio, CEC = cation exchange capacity.

## Discussion

### Cover soil selection

Direct placed forest topsoil derived from coarse texture soil provides a valuable source of propagules and soil that supports boreal forest plant community development. Our results align with other studies that show forest topsoil having greater species richness, diversity and desired forest species cover and density than peat-mineral substrate [5, 7]. Forest topsoil provided tree and shrub densities greater then the common planting prescription of 2500 woody stems per ha [38]. After three years, shrub densities on forest topsoil were similar to densities in naturally disturbed and partially harvested upland forest stands [39, 40]. The greater densities of dryland plants and canopy cover using forest topsoil is due to dryland species being adapted to the drier reclaimed landscapes, and these species are already present within the soil propagule bank. Few pioneer species contributed to the majority of densities for total, forb, grass and sedge plant groups in peat-mineral treatments; the lack of density differences does not reflect the large difference in species richness between the two treatments. The increase in species richness in peat-mineral treatments is largely attributed to upland species egress from forest topsoil treatments. Our results further support findings from Jones and Landhäusser [7] where patches of forest topsoil helped establish more species on adjacent reclaimed land with peat-mineral substrate.

Greater cover with forest topsoil can be attributed to factors other than species adapted to drier landscapes, such as more available phosphorus and lower electrical conductivity and pH relative to peat-mineral substrate. Cover reflects protection plants are contributing against soil erosion, giving a good estimate of ecological significance and reflecting ecosystem function [41]. Electrical conductivity and pH in forest topsoil were more suitable for plants than peat-mineral substrate. Both are rated good in forest topsoil and fair in peat-mineral substrate as per soil quality criteria [42]. Soil pH is an important factor regulating plant growth [43] and if elevated could result in deficiencies of ions unavailable at high pH [44]. Electrical conductivity is an indicator of soil salinity, which can limit plant growth by water imbalance or ionic imbalances resulting in increased energy use [43]. Most boreal species are intolerant of saline soils [45]. Forest topsoil use almost ensures electrical conductivity will be rated as good by soil quality criteria [42], because there are few naturally saline areas in the mineable oil sands region [45].

Greater total carbon, total nitrogen, electrical conductivity, pH and cation exchange capacity in peat-mineral substrate were reported in other studies [5, 46–48] from mixing peat with over stripped, alkaline mineral soil [1]. Peat-mineral substrate often has less available and exchangeable potassium and phosphorus than forest topsoil [5, 47, 48]. Available phosphorus is limiting in boreal forest soils [49]. Using forest topsoil developed on coarse texture soil could reduce the need for phosphorus and potassium fertilizer.

### Salvage depth

Seed density, root abundance and species richness decrease with depth in natural soils and our propagule bank study confirmed this; however, shallow and deep salvages were similar in species richness. Results in our study for species richness contradict the few studies of salvage depth effects on plant establishment. Shallow salvage generally resulted in increased species richness in our study, but not statistically significant. Rokich et al. [27] found salvaging 10 cm of surface soil from *Banksiana* woodland increased (22.0 vs 15.7) species from 30 cm salvage. Tacey and Glossop [50] found salvaging 5 cm of surface soil from jarrah forest increased species richness (42 vs 35) relative to 40 cm salvage. Fair [51] found 23 native boreal species on topsoil salvaged at 15 cm and 19 species salvaged at 40 cm. We had a difference of 15 cm between salvage depths whereas other studies had differences of at least 20 cm [25, 27, 50, 51]. The deep salvage might not have been deep enough to dilute the propagule bank that would reduce the number of plants and species establishing from the in situ propagule bank in the donor soil. We found most species established from vegetative propagules, which could explain the few differences in diversity given roots are found deeper within the soil profile relative to seeds. If soils were salvaged below 25 cm, a threshold would likely be obtained and shallow salvage would result in establishment of more species in greater abundance.

Greater densities for all plant groups with shallow salvage were expected; however, small or non-significant effects of salvage depth were found for herbaceous and sedge groups. Greater tree, shrub, grass and lily densities from shallow salvage were not surprising considering deep salvage would dilute propagules in forest topsoil. Fair [51] found salvaging topsoil on fine texture soil at 15 cm increased plant group densities relative to 40 cm salvage. Rokich et al. [27] found greater species recruitment on a bauxite mine when soil was salvaged at 10 cm (254 seedlings in 5 m^2^) than 30 cm (81.33 seedlings in 5 m^2^). Tacey and Glossop [50] found stripping 5 cm of topsoil significantly increased seedling establishment relative to stripping 40 cm in jarrah forest. Lack of significant differences between salvage depths could be attributed to factors reducing emergence with shallow salvage such as soil temperature, soil water or propagule to soil contact. Shallow salvaged forest topsoil contained more roots and organic matter and less sand, which could lead to less available water and soil contact for seed germination and emergence from propagules. Further research is needed since only 25 cm salvage was studied and with soils salvaged too deep a threshold could be reached resulting in few plants because of dilution.

The difference in plant density response to salvage depth in this experiment might also be explained by increased variability with large plot sizes and equipment for soil handling. In other experiments [27, 50], salvage areas and plot sizes were much smaller, and smaller equipment was used. Salvaging soil from large areas with large equipment reduces precision. Placement of deep salvaged soil containing large roots with large equipment did not mix forest floor layers and mineral soil well. However, using larger plots and equipment presents both unique opportunities and challenges. The main advantage is the ability to cover larger areas, potentially leading to more comprehensive and ecologically relevant data. This comes with the trade-off of reduced precision and challenges in soil mixing. Constructing plots at a large scale provides a more realistic representation of field conditions, unlike very small controlled plots, which may not properly represent field conditions.

Few studies have assessed effects of salvage depth on plant cover. Increased cover with shallow salvage would be expected as shallow salvage contained more organic matter and plant available nutrients. Shallow salvage resulted in higher canopy covers for most plant groups and greater organic carbon and total nitrogen. A combination of shallow salvage and deep placement typically resulted in the greatest cover. Shallow salvage and deep placement had greater available macro nutrients than other forest topsoil treatments on sand and to a lesser extent on peat-mineral substrate. Increased soil organic carbon and nutrients with shallow salvage and deep placement help explain the greater cover. Shallow salvage better maintains organic carbon and macro nutrients than deep salvage which can dilute the nutrient rich forest floor layer.

Salvage depth impacts soil physical, chemical and biological properties which can affect how forest topsoil should be placed for reclamation. Distribution of organic matter and nutrients required for plant growth decreases lower in the natural soil profile [52, 53]. However, in this research, available phosphorus increased with deep salvage. The donor site Bm horizon had more available phosphorus than Ae (data not shown), thus available phosphorus increased with deep salvage. Lanoue [54] found high phosphorus in B horizons in jack pine forests on coarse texture soils. Salvaging forest topsoil developed on coarse texture soil would provide an increase in phosphorus; however, soil organic matter and other nutrient concentrations would decrease.

Recommending one salvage depth for all soil types might not be ideal to optimize forest topsoil. Different plant communities could require different amounts of soil nutrients and organic matter to maintain productivity. Expectations can differ for diversity. For example, deep (20 to 30 cm) salvage increases volume of material for reclamation; however, increased depth limits suitability as a propagule source for revegetation and could reduce organic matter. Placing shallow salvaged (10 to 15 cm) forest topsoil on selectively salvaged subsoil with the intent of creating biomass might not use forest topsoil efficiently. Subsoil provides additional nutrients and using both materials means less available material for reclamation. These examples demonstrate different approaches for managing and using salvaged forest topsoil. Shallow salvage should be targeted when reclaimed site productivity, and to a lesser extent, species diversity are primary objectives. Deep salvage should be targeted when the primary objective is obtaining maximum reclamation material volume.

### Placement depth

Most studies found deep placements (30 to 60 cm) did not increase species richness or diversity and shallow (10 to 15 cm) placements often resulted in increased values [27, 55, 56]. Our results were most similar to those of Holmes et al. [26] who found slight differences in species richness between different placement depths. While deeper placements had slightly more species there were periods where shallow placements had more. Density of most plant groups did not differ between placement depths and trends were similar to those for species richness; however, deep placement favoured higher densities of shrubs on the sand substrate. Waryszak et al. [57] found deeper placement depths increased *Banksia* woodland species richness and emergence relative to shallow placement. Increased shrub densities on deeper placement depths could result in less propagule deterioration (e.g. friction) resulting in a better quality seeding mix [57].

Deeper placement generally results in greater plant cover and/or productivity [55, 58–60]. Archibald et al. [28]) found greater vegetation cover and richness on 20 cm cover soil placements. Holmes et al. [26] found cover of unfertilized plots was greater with 30 cm of topsoil than 10 and 0 cm on a South African mine. Differences between placement depth were greater over time. Bowen et al. [56] found that in south central Wyoming over 24 years, deeper placement resulted in increased grass cover; however, forb cover was greatest with no topsoil. Grass cover was significantly greater with 40 cm of topsoil than 0 and 20 cm, but not different than 60 cm. They attributed forb cover increase with shallow placement to less competition from grasses. Fair [51] found topsoil salvaged and placed at 15 cm resulted in a significant cover increase for most functional plant groups than to topsoil salvaged and placed at 40 cm, attributed to less dilution of the propagule bank. After three years topsoil placed at 40 cm had similar native plant species cover to 15 cm depths [25].

If placement depth is shallow, available nutrients might not be sufficient for plants to respond with increased cover. It is not surprising deep placement of forest topsoil on a nutrient poor substrate, such as sand, would result in greater cover considering there is more available nutrients and organic matter than with shallow placement. MacKenzie and Naeth [5] assessed effects of placement depth of two surface soils on a saline-sodic overburden dump and found significant interaction effects with cover soil type and placement depth. Forest topsoil from fine texture surface soil placed at 20 cm had greater cover of all vascular plants than 10 cm placement. However, cover was not different between 20 and 10 cm placements with peat-mineral substrate.

Placement depth should be based on reclamation objectives and optimal use of material if quantities are limited. Optimal placement depth of forest topsoil to sustain a mature, productive forest could be different than depth for diverse wildlife habitat. Important considerations for reclaiming productive forests are available soil water and growing space for tree roots [61]. Deep soil positively influenced mine soil productivity through increased rooting depth and greater soil water retention [62, 63]. Topsoil placement for a less productive forest plant might be shallower than that for commercial forest. For increased species diversity, placement should be varied from shallow to deep [64]; however, if propagules are buried too deeply they could lie dormant and lose viability, or germinate but never establish. If soil is applied at shallow depths, propagules can emerge but available water and nutrients could limit plant establishment. Application of shallow soil layers over substrates with adverse properties (salinity, sodicity) requires further research. Initial growth might appear successful; but over time vigour could decrease as salts ingress into overlying soil.

### Substrate considerations

Key determinants affecting plant establishment and growth are species requirements, substrate quality, annual precipitation and quality and depth of replaced soils [65, 66]. Where underlying substrate has adverse characteristics for root growth, depth of soil replaced depends on nature and severity of the substrate, increasing with severity of adverse properties [65]. This can explain placement depth having more effect on sand than peat-mineral substrate. Fewer cover differences for most plants with forest topsoil on peat-mineral substrate could result from substrate providing high organic matter, allowing plants in shallow placement to access more water and nutrients.

Effects of salvage and placement depth on chemical properties varied with substrate, with shallow salvages on sand generally having highest values of most properties. Fewer significant differences in macro nutrients were detected between salvage depths on peat-mineral substrate than sand and organic carbon and available nutrients were lower in forest topsoil on sand. Shallow placed forest topsoil on peat-mineral substrate had soil properties similar to those of peat-mineral substrate. More nutrients with shallow placement can be attributed to higher concentrations in substrates; however, reduced nutrient uptake from lower plant productivity could be a factor. MacKenzie and Naeth [5] found admixing increased with shallow forest topsoil or peat-mineral applications, causing a change in soil chemistry with topsoil more similar to that of the substrate.

Deep placement of forest topsoil on nutrient poor substrate, such as sand, would result in greater cover considering there is more available nutrients and organic matter than with shallow placement. MacKenzie and Naeth [5] found significant interaction effects with cover soil type and placement depth of two surface soils on a saline-sodic overburden dump. Forest topsoil on fine texture surface soil placed at 20 cm resulted in greater cover for all vascular plant groups than 10 cm placement. Cover was not different with 20 and 10 cm placement using peat-mineral substrate. If placement is too shallow on nutrient poor substrates available nutrients might not be sufficient for plants to respond with increased cover.

Greater canopy cover on multiple treatments on peat-mineral substrate is attributed to mixing peat-mineral and forest topsoil during placement. The chemistry of peat-mineral substrate underlying forest topsoil would influence topsoil surface soil chemistry. Shallow placement of forest topsoil on substrates with more organic matter, nutrients and soil water retention could help reduce the need for deep applications of forest topsoil. Where subsoil properties are not limiting, topsoil amount and quality becomes less important [67]. Long term effects on plant community establishment from placing topsoil developed on sandy parent material with low organic carbon on a substrate that has more organic carbon is unknown. Increased soil water retention on peat-mineral substrate could shift a *Pinus banksiana* stand to mixed *Pinus banksiana* and *Populus tremuloides*. Plants in shallow forest topsoil on peat-mineral substrate would be more influenced by substrate properties than those on deep forest topsoil. For example, electrical conductivity was significantly greater with shallow placement on peat-mineral substrate. Caution should be taken layering topsoil over substrates that are deficient or harmful, because negative shifts in plant community could occur.

## Conclusions

Forest topsoil on coarse texture upland surface soils developed under *Pinus banksiana* forests provides a rich source of seeds and plant propagules for revegetation; many of these species are not commercially available. Forest topsoil provides an alternative cover soil that can initially support an early successional plant community. Salvaging to 25 cm likely did not reach a dilution threshold to see significant reductions in plant density or diversity; however, shallow salvage had greater tree stem densities. Shallow salvage often resulted in higher canopy cover for most plant groups; however, responses were species specific. Deep placement had little effect on plant density for most plant groups and generally resulted in greater canopy cover. A balance between maximizing the area over which propagules are redistributed, while providing sufficient resources for successful plant establishment is needed. If adequate diversity in plant communities is a reclamation goal, topsoil could be applied at shallower depths than those to maximize total diversity. When forest topsoil was applied to peat-mineral substrate, there were fewer differences between shallow and deep salvage in the resulting canopy cover and multiple treatments had greater cover. Further research is needed to assess the long-term impacts of different salvage and placement depths on plant community development and to investigate the potential effects of varying substrate properties on soil and plant response in reclaimed oil sands landscapes.

## Supporting information

S1 Dataset Cover and diversity data. (XLSX)

S2 Dataset Plant density data. (XLSX)

S3 Dataset Soil data. (XLSX)
